# Influence of Contoured Insoles with Different Materials on Kinematics and Kinetics Changes in Diabetic Elderly during Gait

**DOI:** 10.3390/ijerph191912502

**Published:** 2022-09-30

**Authors:** Qiu-Qiong Shi, Pui-Ling Li, Kit-Lun Yick, Jiao Jiao, Qi-Long Liu

**Affiliations:** 1 School of Fashion and Textiles, The Hong Kong Polytechnic University, Hong Kong, China; 2 Laboratory for Artificial Intelligence in Design, Hong Kong, China; 3 Dr. Stephen Hui Research Centre for Physical Recreation and Wellness, Hong Kong Baptist University, Hong Kong, China

**Keywords:** contoured insoles, diabetic foot, gait pattern, insole materials, peak plantar pressure

## Abstract

Background: Alterations in the lower limb kinematics and kinetics of diabetic patients have been reported in previous studies. Inappropriate choices of orthopedic insole materials, however, fail to prevent diabetic foot ulcers and modify abnormal gait. The aim of this study was to quantitatively compare the effects of contoured insoles with different materials on the kinematics of and kinetics changes in the diabetic elderly during gait. Methods: There were 21 diabetic patients who participated in this study. Three-dimensional (3D) experimental contoured insoles constructed of soft (i.e., Nora Lunalastik EVA and PORON^®^ Medical 4708) and rigid (i.e., Nora Lunalight A fresh and Pe-Lite) materials with Langer Biomechanics longitudinal PPT^®^ arch pads were adopted. An eight-camera motion capture system (VICON), two force plates, and an insole measurement system—Pedar^®^ with 99 sensors—were utilized to obtain the kinematics and kinetics data. The plug-in lower body gait model landmarks were used for dynamic data acquisition during gait. The corresponding data from five gait cycles were selected and calculated. Results: The range of motions (ROMs) of the ankle joint (*p* = 0.001) and knee joint (*p* = 0.044) were significantly influenced when the contoured insoles were worn in comparison to the barefoot condition. The joint moments of the lower limbs with maximum ankle plantarflexion during the loading response and maximum knee and hip flexions were significantly influenced by the use of contoured insoles with different materials in the diabetic elderly. The peak plantar pressure (PPP) of the forefoot (*p* < 0.001), midfoot (*p* = 0.009), and rearfoot (*p* < 0.001) was significantly offloaded by the contoured insoles during the stance phase, whilst the PPP of the rearfoot (*p* < 0.001) was significantly offloaded during the swing phase. Conclusions: The contoured insoles, especially those constructed with soft materials, significantly offloaded the PPP during gait—hence accommodating certain abnormal gait patterns more effectively compared to going barefoot.

## 1. Introduction

Diabetes is a chronic disease and was responsible for 6.7 million deaths in 2021 worldwide [1,2]. Diabetic patients are also at higher risk of falls during walking—especially the elderly—when compared to healthy individuals [3,4]. Walking is an essential component of physical activity for daily life. However, dysfunction of the intrinsic foot muscles has been observed in diabetic patients, which leads to increased gait disorders [5]. Numerous previous studies have reported changes in the lower limb kinematics and kinetics of diabetic patients, including a reduced range of motion at both the ankle [5,6,7,8] and knee joints [9,10]—which are associated with step and stride lengths—and double support time in diabetic patients [11]. The results of kinetics data from diabetic patients are more debatable. For instance, Sacco et al. [12] found that a diabetic group experienced increased hip flexion moment at push-off and decreased extensor moment at initial contact compared to a non-diabetic group during walking; however, opposite results were reported by Fernando et al. [10].

These biomechanical alterations in gait are associated with plantar pressure changes [8,13]. Peak plantar pressure (PPP) is used as a measure of trauma to determine the initiation of diabetic foot ulcers (DFUs) [13]. Higher plantar pressures are considered to be a key risk factor for DFUs [14] and long-term exposure to abnormal plantar pressure during daily activity more readily causes gait disorders. Previous studies have claimed that higher PPP values are found in the forefoot and midfoot of diabetic patients when compared to groups without diabetes [9,10,15]; however, Fernando et al. [10] also found significantly higher PPP in the rearfoot of diabetics. Due to the higher PPP during gait that is associated with DFUs [16,17], and simultaneously the increased risk of DFU morbidity and gait disorder in diabetes [15], it is essential to reduce the PPP through various intervention to prevent DFUs and gait alteration—especially for the diabetic elderly, who are at higher risk of falling than age-matched control subjects [18].

Orthotic devices (e.g., orthopedic shoes, orthoses, or insoles) are prescribed and considered the most common intervention for DFU treatment to offload the PPP during walking [13,15,19]. Custom-fabricated orthopedic insoles are engineered to modify the kinematics and kinetics of locomotion and to control gait stability, facilitate shock absorption, and prevent the formation of DFUs [20,21,22]. The structural design and material properties of these insoles are particularly essential and are related to the efficacy of foot orthotic interventions for DFUs and gait alteration. With advances in materials sciences in recent years, a wide range of materials have been introduced to the market, such as ethylene vinyl acetate (EVA; e.g., Nora^®^ (Weinheim, Germany)), polyethylene (e.g., Pe-Lite^®^ (Algeos, Liverpool, UK)), and polyurethane (e.g., PORON^®^ (Dr. Jill’s Foot Pads, London, UK)) [23,24,25,26]. These insole materials are available in a wide range of hardnesses, thicknesses, and densities, with different structural and mechanical properties for a diversity of different usages. Such insole materials can be divided into rigid and soft for different applications and end-uses. Previous studies have claimed that rigid materials have better supporting qualities, but with lower tolerance to wear [24,27]. Soft insole materials have been found to protect vulnerable tissues from trauma during daily activities but might not treat the underlying DFUs or correct the gait disorder [20]. Besides this, compared with flat insoles, three-dimensional (3D) contour insoles with arch supports show better performance in offloading the plantar pressure of diabetic feet—as indicated in previous studies [28,29,30,31].

Although the effects of insoles on plantar pressure have been well documented, there is still insufficient evidence on selecting the type of insole materials for optimal plantar pressure offloading and abnormal gait modifications [32]. Inappropriate insole materials cannot prevent DFUs nor change abnormal gait in the diabetic elderly. In addition, most previous studies have focused on the insole effect on offloading plantar pressure—yet studies on the effect of insoles on gait analysis have been limited [15,23,26,28,29]. In diabetic patients, alterations in gait features are related with plantar pressure changes [33]. Therefore, the aim of this study is to quantitatively compare the effects of the above-mentioned different insole materials on plantar pressure offloading and abnormal gait modification during both the stance and swing phases during gait in the diabetic elderly. We hypothesize that the type of insole material not only has a major influence on the offloading performance and PPP reduction during gait, but also modifies gait abnormalities.

## 2. Methods

### 2.1. Participants

Human subject ethics approval was granted by the University Ethics Committee (HSEARS20200128001). The inclusion criteria were: (a) age ≥ 50 years old and (b) diagnosis of Type 1 or 2 diabetes. The exclusion criteria were: (a) lower extremity amputation, (b) active or history of DFU, (c) shuffling gait [5] or inability to walk 10 m without assistance [5,34,35], and (d) blood pressure higher than 130/90 mmHg [36]. The sample size was calculated by using G * power (Kiel, Germany) for a two-tailed test with 1 − β = 0.08 and α = 0.05 with five groups. Twenty-one (21; 12 males and 9 females) diabetic patients (age: (mean ± SD) 62.3 ± 5.5 years old; height: 164.2 ± 6.2 cm; body mass: 65.1 ± 11.6 kg; body mass index: 24.1 ± 3.5 kg/m^2^; duration of diabetes: 9.2 ± 8.4 years; distribution of diabetes: Type 1 (8 participants), Type 2 (13 participants); shoe size: EU 39.2 ± 1.9 (female), EU 41.4 ± 1.0 (male)) were recruited for this study. The participants signed a consent form prior to the experiment after a briefing of the procedures.

### 2.2. Materials

**Figure 1:**
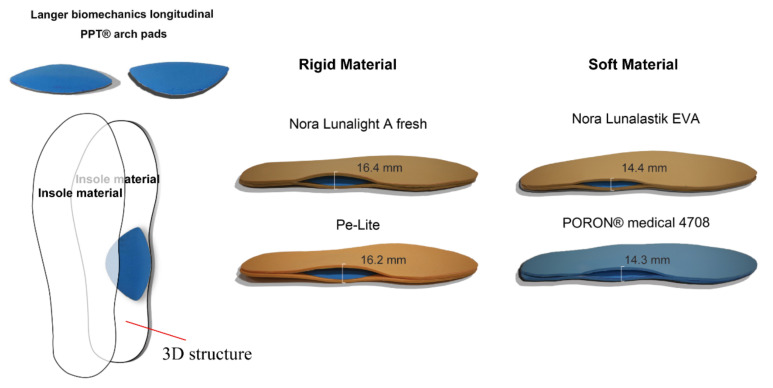
Contoured 3D insole structures with different materials.

Each experimental contoured insole was cut out from either a soft or rigid material (see Table 1) and fabricated into a three-dimensional (3D) structure: a 2-layer insole material inserted with a Langer Biomechanics longitudinal PPT^®^ arch pad (Langer Biomechanics, Mesa, AZ, USA) between each layer (Figure 1). The same type of leather shoes (Kinghealth, Hong Kong) were given to each participant based on their shoe size during the experiment.

### 2.3. Experimental Protocol

Each participant changed into tightly fitting clothing when they arrived at the laboratory to minimize the movement artifacts resulting from loose clothing [37]. Then, the Pedar^®^ insole measurement system (Novel GmbH, Munich, Germany) with 99 sensors of the appropriate size were inserted into the shoes and placed on the top of the insoles, or into the socks for plantar pressure measurement with or without insoles during gait. The data collection frequency of the Pedar^®^ was 50 Hz per second. Then, sixteen passive-reflective markers of 14 mm in diameter were placed on the participant according to the landmarks set in the plug-in lower body model [38]. An 8-camera motion capturing system (VICON, Nexus 2.0 Inc., Oxford, UK) and 2 force plates (AMTI, Advanced Mechanical Technology, Inc., Watertown, MA, USA) mounted under the walkway were utilized for gait analysis and recorded 100 frames per second simultaneously. The Pedar^®^ system was synchronized with the VICON and 2 force plates during the experiment. All of the systems were calibrated before the experiment.

Then, the participant walked on the force plate at a self-selected walking speed to perform his/her normal gait [39] and the foot would land on the force plate naturally [40]. The average walking speed was 0.9 ± 0.2 m/s. Each participant repeated walking trials until a minimum of 5 “clean” foot force plate contacts with both the right and left limbs were acquired [37,41]. The corresponding data from 5 gait cycles were selected to calculate the plantar pressure distribution and gait analysis under different insole material conditions. Each condition was conducted in a randomized order.

### 2.4. Kinematics and Kinetics Data Acquisition

The gait analysis included kinematics and kinetic data collection and a comparison of different conditions. The duration of one gait cycle was defined as the interval between 2 consecutive heel contacts of the same foot [42]. The kinematics movements of the participants were captured with reflective markers. The ankle, knee, and hip joint angular displacements and their range of motion (ROM) were calculated for each participant and compared based on the different insole material conditions and in the barefoot condition.

**Figure 2:**
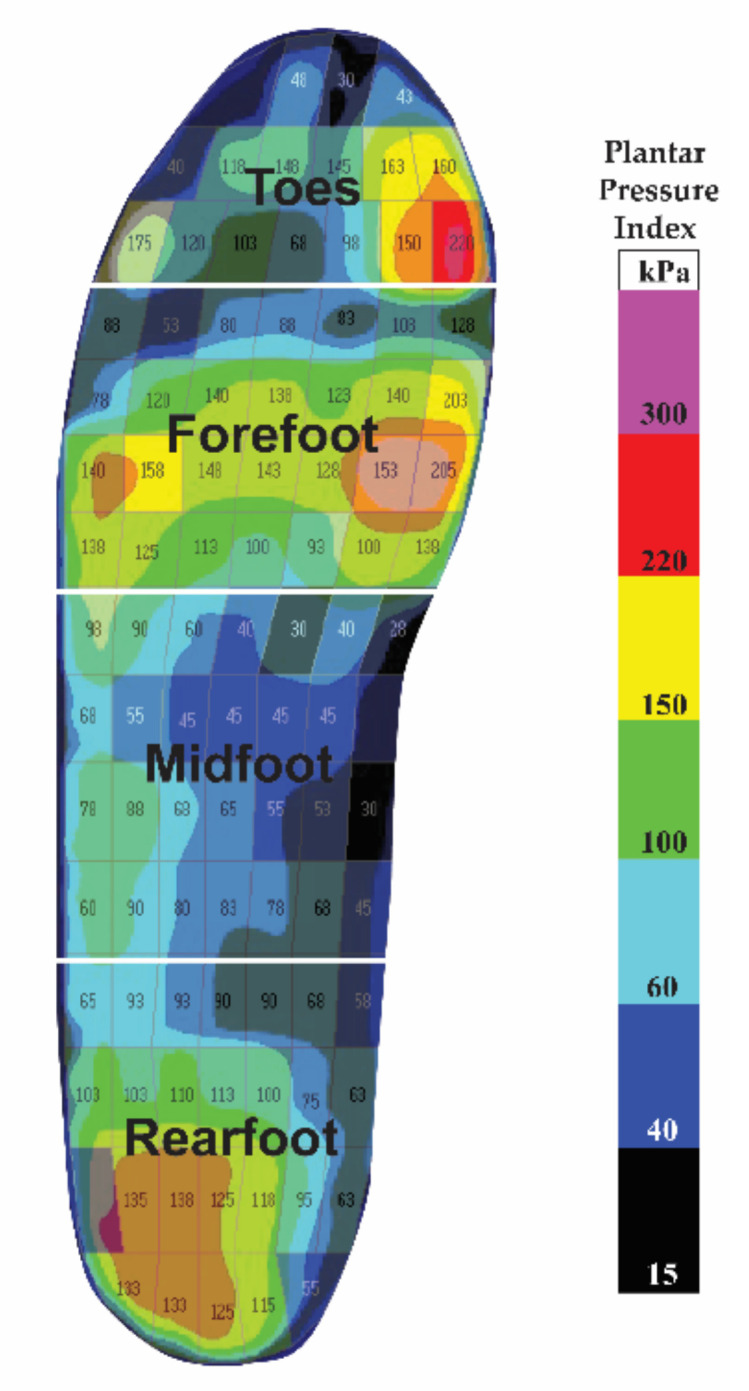
Four specific plantar regions.

The kinetics data were calculated by using the force generated during the stance phase. Moment and power parameters were normalized to the weight of the patients [41]. All of the kinematics and kinetics data were interpolated into a 101 data point length for each gait cycle. The plantar was divided into 4 specific regions including the toes, forefoot, midfoot, and rearfoot (Figure 2). The maximum peak pressure in the four specific foot regions during different gait cycle phases (i.e., stance and swing) were selected and compared based on the different insole materials or in the barefoot condition. The plantar pressure data of each gait cycle consisted of 60% stance (i.e., from heel strike to toe off) and 40% swing phase (i.e., from after toe off to heel strike) [13] to ensure that each gait cycle consisted of a 101 data point length.

### 2.5. Statistical Analyses

SPSS software (SPSS Statistics IBM, Version 20.0, Chicago, IL, USA) was used to test for statistical analysis. A Student’s *t* test was first applied to see if significant differences existed between the dominant and non-dominant feet in plantar pressure and gait pattern after normal distribution testing. If no significant difference was found between the feet, a one-way repeated measure ANOVA was employed to examine the insole effects within the groups of the dominant foot. *p* values of less than 0.05 were set as statistically significant. If significance was found, post hoc testing with a Bonferroni correction was used to identify the difference between the conditions. All of the data were presented as mean and standard deviation (SD; i.e., mean (SD)).

## 3. Results

There was no significant difference between the dominant or non-dominant foot in either kinematics (*t*_216.234_ = 0.435, *p* = 0.442) or kinetics (*t*_216.359_ = 1.271, *p* = 0.326) for the diabetic elderly during gait. Therefore, the following results show the insole effects within the groups in the dominant foot.

### 3.1. Effects of Insole Materials on Kinematics

**Figure 3:**
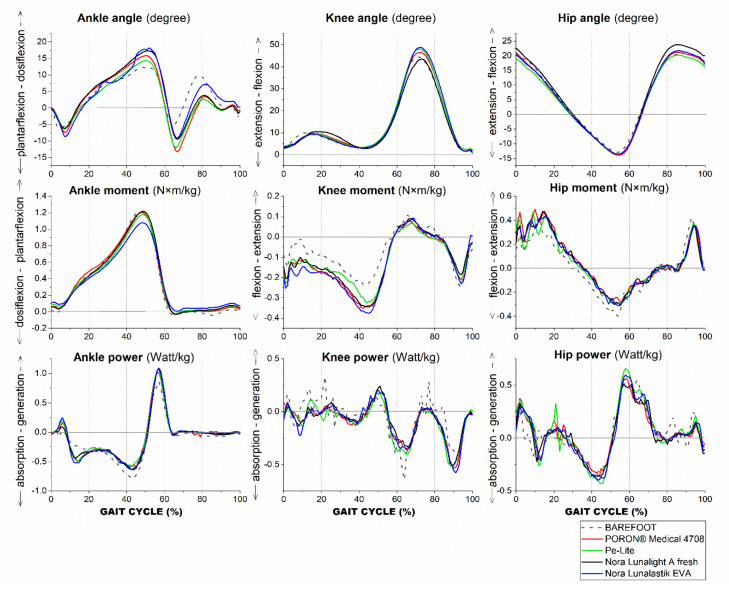
Mean of kinematics and kinetics of the ankle, knee, and hip: 5 conditions during gait.

Table 2 shows that the ROMs of the ankle joint (*F*(1.694,33.889) = 10.336, *p* = 0.001, $\eta_{p}^{2}\;$= 0.341) and knee joint (*F*(2.430,48.594) = 3.119, *p* = 0.044, $\eta_{p}^{2}\;$= 0.135) were significantly increased when insole materials were worn compared with the barefoot condition. The differences were most pronounced at the ankle joint (see Figure 3). Pairwise comparisons showed that the ROMs of the ankle joint were significantly increased by four experimental insoles (*p* < 0.05) during gait.

Figure 3 shows that the participants walked with higher maximum dorsiflexion and plantarflexion during the stance phase with an insole compared with the barefoot condition. The maximum knee flexion was significantly influenced by the insole material (*F*(2.712,54.244) = 3.442, *p* = 0.027, $\eta_{p}^{2}\;$= 0.147), and especially was significantly higher with the Nora Lunalastik EVA (*p* = 0.044) versus barefoot, while the maximum knee extension was slightly higher in the barefoot condition. The maximum hip extension showed smaller values and the maximum hip extension occurred earlier in the barefoot condition than with the use of an insole during gait (see Figure 3).

### 3.2. Effects of Insole Materials on Kinetics

Table 3 shows the changes in the kinetics with different conditions. The ankle moment of the maximum plantarflexion and dorsiflexion were greater in the barefoot condition as compared to the use of the four insoles. The maximum ankle power generation deteriorated during stance and the absorption during the loading phase was reduced in the barefoot condition.

At the knee, the maximum extension moment during stance in the barefoot condition was slightly higher than that with the insoles donned—including the use of PORON^®^ Medical 4708, Pe-Lite, and Nora Lunalastik EVA. The maximum flexion moment was significantly influenced by the insole materials in the single support phase (*F*(4,84) = 3.018, *p* = 0.022, $\eta_{p}^{2}\;$ = 0.126). The use of PORON^®^ Medical 4708 significantly increased the maximum flexion moment (*p* = 0.032) compared to the barefoot condition. Through initial contact to toe-off, the knee extension moment was greater in the barefoot condition than in the four insole conditions (see Figure 3). The maximum absorption during stance in the barefoot condition was also higher than that in the contoured insole conditions (*F*(1.782,37.422) = 3.470, *p* = 0.046, $\eta_{p}^{2}\;$ = 0.142). More power was generated during single support in barefoot than with the use of insoles, while spikes were frequently shown in the barefoot condition during the gait cycle (see Figure 3).

At the hip, the maximum extension moment was slightly higher in the barefoot than in the four insole conditions. Except for Pe-Lite, the maximum flexion moment was significantly higher in the barefoot (*p* < 0.05) than with the use of the experimental insoles. Although the differences in maximum power generation and absorption during stance among the five conditions were small, repeated spikes could be found in the barefoot condition during gait.

### 3.3. Effects of Insole Materials on Offloading PPP Values

**Figure 4:**
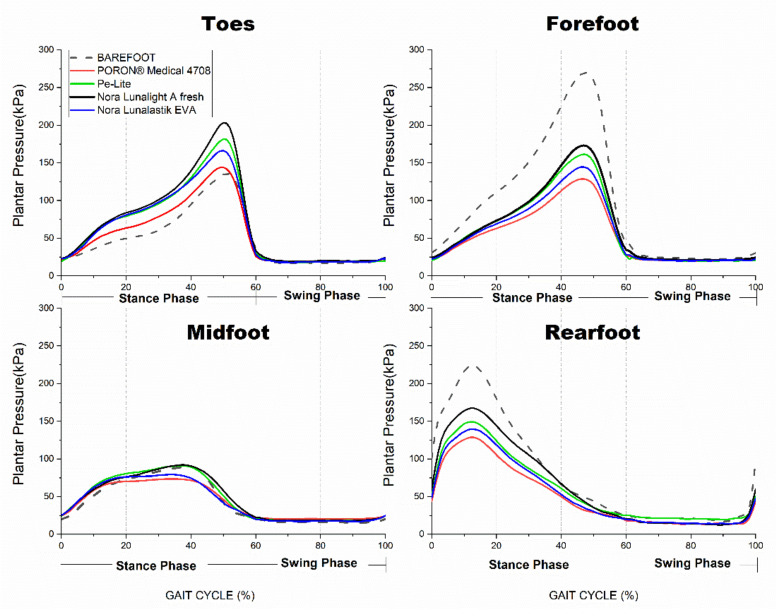
Mean of plantar pressure in four specific plantar regions for 5 conditions during gait.

Table 4 shows that the performance of PPP offloading in the four plantar regions with different conditions. During the stance phase, PPP offloading was significantly affected in the four plantar regions by the insole materials (*p* < 0.05). However, the pairwise comparisons showed that the four types of insole materials significantly lowered the PPP values in the forefoot and rearfoot only (*p* < 0.001) when compared with the barefoot condition. Regardless of the plantar region, the PPP values were the lowest with the use of PORON^®^ Medical 4708 insole during stance among the five conditions—particularly in the forefoot and rearfoot (*p* < 0.001) from heel strike to toe-off (see Figure 4).

During the swing phase, the experimental insole material only significantly offloaded the PPP values in the rearfoot (*p* < 0.001). Compared with the barefoot condition, the four insole materials significantly lowered the PPP values in the rearfoot (see Table 4). At the peak value point during the terminal swing phase, the PPP value with PORON^®^ Medical 4708 was the lowest in the rearfoot.

## 4. Discussion

To the best of the knowledge of the authors, this is the first study that has quantitatively analyzed the effects of a contoured insole made of different materials on kinematics and kinetics changes during stance and swing phases in the diabetic elderly. The main findings demonstrated that the four experimental insole materials had a significant effect on abnormal gait modifications, whilst significantly offloading the PPP in the plantar regions of the forefoot, midfoot, and rearfoot during stance, and in the rearfoot during swing. The findings show evidence to support our initial hypothesis.

The diabetic elderly subjects walked with significantly increased ROMs of the ankle and knee joints when they wore the contoured insoles compared to the barefoot condition in this study. The ankle ROMs were especially and significantly increased with the use of the contoured insoles with four types of materials during gait. As compared to the barefoot condition, the soft insole material of PORON^®^ Medical 4708 showed around a double increase in maximum ankle plantarflexion. This aligns with Gomes et al., who found that diabetics showed reduced ROMs in their ankle and knee joints compared with a healthy group [43]. Ko et al. also confirmed that diabetes had reduced ROMs in the ankle during self-selected speed walking [44]. The experimental insoles also showed a significant influence on maximum knee flexion. The use of a soft insole (Nora Lunalastik EVA) resulted in significantly greater maximum knee flexion at toe-off. A previous research work demonstrated that diabetics walk with reduced knee flexion when compared to other healthy peers [43]. This abnormal kinematics pattern in the diabetic elderly was modified by wearing the contoured insoles in this study.

The contoured insoles were found to significantly influence the joint moments of the lower limbs with maximum ankle plantarflexion during the loading response as well as the maximum knee and hip flexions. The maximum knee flexion moment was significantly increased when a soft insole (PORON^®^ Medical 4708) was worn versus the barefoot condition. The maximum knee extension moment was higher during barefoot walking compared to three of the insole conditions: soft—PORON^®^ Medical 4708 and Nora Lunalastik EVA, and rigid—Pe-Lite. Sao et al. [12] reported that diabetics show higher values of maximum knee extension moments during early stance. Meanwhile, a significantly higher maximum hip flexion moment was found in the barefoot condition compared to the use of soft—PORON^®^ Medical 4708 and Nora Lunalastik EVA, and rigid—Nora Lunalight A fresh insoles. Sacco et al. [12] showed that diabetics have higher hip flexion moments during gait. The diabetic elderly individuals also exhibited larger maximum hip extension moments in their barefoot condition than with the four contoured insoles in our findings—which is in line with Sawacha et al. [40], who reported a larger hip extension moment in diabetic patients. A significant effect was found for maximum knee absorption from the contoured insoles in this study. The diabetic elderly had less ankle generation and absorption, but higher knee generation and absorption in their barefoot condition than with the four contoured insoles. Ko et al. [44] found that diabetics have higher knee absorption with lower ankle generation during gait. Aligning with Henderson’s findings, the decreased ankle power generation in diabetics during terminal stance tended to associate with weakness in the distal muscles, whilst there was a compensatory increase in knee and hip power generation [45]. Meanwhile, many spikes could be observed in both the knee and hip joint power during gait in the barefoot condition. Yick and Tse [20] claimed that spikes represent a less-smooth acceleration in knee and hip power generation and absorption. They also found that insoles can absorb the energy of heel strikes while protecting the joints from the rebounding spikes of force by gradually and evenly dissipating energy [20]. Therefore, from the aforementioned evidence, it can be deduced that the diabetic elderly exhibited more efficient gait patterns when they donned one of the four contoured insoles—especially those with soft insole materials—in this study.

Modifications in biomechanical gait patterns are related to altered plantar pressure. Significantly lower PPP values could be observed in the forefoot and midfoot at toe-off during stance with the use of the contoured insoles when compared to the barefoot condition. While significantly lower PPP values in the rearfoot were found at initial contact during stance and terminal swing, the soft insole material (PORON^®^ Medical 4708) resulted in the optimal plantar pressure offloading in the forefoot and rearfoot from the initial contact to the terminal stance amongst the four studied insole materials. The time points of the PPP in the forefoot and midfoot during stance happened before or close to these when the maximum ankle plantarflexion and maximum knee flexion occurred during gait in this study. Sacco et al. [8] found that diabetic patients have reduced active ankle ROM with higher PPP values in the forefoot and midfoot at the push-off phase when compared with a healthy age-matched group. It is known that elevated plantar pressure can be prevented in diabetic patients by increasing the ROMs of the lower limb joints during gait [33]. Thus, it can be concluded that the diabetic elderly exhibited larger degrees of freedom of the distal joints associated with significant plantar pressure offloading in the forefoot and midfoot with the contoured insoles in this study.

Compared with the two rigid insoles (i.e., Pe-Lite and Nora Lunalight A fresh), the ROMs of the ankle and knee joints were higher with the soft insole materials (i.e., PORON^®^ medical 4708 and Nora Lunalastik EVA) during gait. Lo et al. [46] found that the rearfoot angle was significantly increased when soft slippers were donned as opposed to the barefoot condition. Eng et al. [47] proved that soft orthotics increased the ROM of the knee joints at the contact and mid-stance phases during running. However, very few studies have studied the effects of rigid and soft insole materials on kinematics and kinetics during gait. Meanwhile, we found that the soft insole materials used in a contoured 3D structure showed a better performance in offloading the plantar pressure in the forefoot, midfoot, and rearfoot during stance—thus further increasing the reduction in PPP from 27.4% to 48.6% of the barefoot PPP (142.3–350.5 kPa). This is in line with Goske et al. [48], who found that plantar pressure reduction was increased by increasing the thickness of the flat PORON insole during gait. Thus, our main findings showed that DFUs in diabetic patients can be prevented through gait improvement and increasing the offloading of the PPP by wearing 3D contoured insoles constructed of soft materials. It is noteworthy that the insole offloading performance could be diminished by repeated cycles of loading or unloading during gait, resulting in the shape or structural deformation of the insoles with prolonged use [20]. The long-term effects of the insoles shall be further studied.

## 5. Conclusions

The contoured insoles, especially those constructed with soft materials, significantly offloaded the PPP in the forefoot, midfoot, and rearfoot during gait, while modifying parts of abnormal gait patterns compared with the barefoot condition. This is the first study that has comprehensively analyzed the effects of different insole materials on kinematics and kinetics during gait and compared the findings with the barefoot condition. Our findings fill this current research gap and could provide clinical evidence and more information to clinicians for prescribing medical insoles associated with gait training for the diabetic elderly.

## Figures and Tables

**Table 1:** Physical properties of the studied insole materials.

Insole Material (2-Layer)		A	B	C	D	E
Thickness(mm)	Under 0 kPa	8.20	8.00	6.20	6.05	8.20
	Under 50 kPa	8.20	7.95	6.01	5.71	7.05
	Under 150 kPa	8.18	7.88	5.48	4.58	5.53
	Under 200 kPa	8.16	7.79	5.04	3.51	4.48
Density (g/cm^3^)		0.35	0.16	0.23	0.20	
Hardness		Rigid 58 shore A	Rigid 30 shore A	Soft 25 shore A	Soft 18 shore A	

Notes: A—Nora Lunalight A fresh; B—Pe-Lite; C—Nora Lunalastik EVA; D—PORON^®^ Medical 4708; and E—Langer Biomechanics longitudinal PPT^®^ arch pads.

**Table 2:** Statistical results for kinematics parameters with and without insoles (mean (SD)).

Parameter	Barefoot	PORON^®^ Medical 4708	Pe-Lite	Nora Lunalight A Fresh	Nora Lunalastik EVA	*p* Value	$\eta_{p}^{2}$
		(Soft)	(Rigid)	(Rigid)	(Soft)		
Ankle angle (degree)							
Max dorsiflexion	13.93 (5.0)	16.08 (3.8)	15.54 (4.7)	17.80 (5.0)	18.60 (10.5)	0.630	0.021
Max plantarflexion	−7.32 (5.9)	−15.11 (12.8)	−14.05 (10.6)	−11.28 (7.4)	−11.73 (14.3)	0.069	0.129
ROM over gait cycle	21.25 (6.5)	31.19 (13.1) ^#^	29.60 (11.0) ^#^	29.08 (10.7) ^#^	30.32 (13.3) ^#^	0.001	0.341
Knee angle (degree)							
Max flexion	45.51 (15.2)	47.50 (18.7)	48.91 (15.8)	44.47 (17.0)	49.79 (17.0) ^#^	0.027	0.147
Max extension	2.30 (6.9)	1.97 (7.3)	1.85 (9.0)	1.52 (8.3)	1.61 (8.4)	0.760	0.017
ROM over gait cycle	44.47 (11.5)	47.57 (13.5)	47.75 (13.1)	44.00 (13.5)	48.93 (13.3)	0.044	0.135
Hip angle (degree)							
Max flexion	22.60 (8.3)	21.98 (9.1)	21.20 (7.7)	24.75 (13.1)	22.59 (7.3)	0.191	0.078
Max extension	−13.47 (6.8)	−15.14 (7.3)	−14.11 (9.6)	−14.75 (11.6)	−14.72 (9.1)	0.771	0.015
ROM over gait cycle	36.07 (8.0)	37.12 (8.4)	35.98 (10.0)	39.50 (8.2)	37.31 (8.5)	0.231	0.070

Notes: **^#^** indicates *p* < 0.05, significant difference from barefoot. Max indicates maximum. ROM indicates range of motion.

**Table 3:** Statistical results for kinetics parameters with and without insoles (mean (SD)).

Parameter	Barefoot	PORON^®^ Medical 4708	Pe-Lite	Nora Lunalight A Fresh	Nora Lunalastik EVA	*p* Value	$\eta_{p}^{2}$
Ankle moment (Nm/kg)							
Max plantarflexion	1.25 (0.3)	1.22 (0.4)	1.14 (0.4)	1.10 (0.4)	1.19 (0.4)	0.112	1.829
Max dorsiflexion in loading response	−0.04 (0.1)	0.01 (0.1)	0.02 (0.1)	0.02 (0.1)	−0.04 (0.1)	0.021	0.127
Ankle power (W/kg)							
Max generation	1.02 (0.4)	1.12 (0.5)	1.15 (0.6)	1.16 (0.6)	1.27 (0.8)	0.317	0.054
Max absorption	−0.38 (0.3)	−0.58 (0.5)	−0.60 (0.7)	−0.53 (0.5)	−0.70 (1.0)	0.111	0.103
Knee moment (Nm/kg)							
Max extension	0.19 (0.2)	0.12 (0.1)	0.12 (0.1)	0.16 (0.2)	0.12 (0.1)	0.115	0.098
Max flexion	−0.36 (0.2)	−0.44 (0.2) ^#^	−0.43 (0.2)	−0.41 (0.3)	−0.43 (0.3)	0.022	0.126
Knee power (W/kg)							
Max generation in single support	0.62 (0.7)	0.35 (0.3)	0.45 (0.4)	0.35 (0.3)	0.47 (0.5)	0.160	0.085
Max absorption during stance	−1.09 (0.8)	−0.61 (0.5)	−0.71 (0.8)	−0.59 (0.3)	−0.70 (0.4)	0.046	0.142
Hip moment (Nm/kg)							
Max extension	0.55 (0.3)	0.46 (0.2)	0.48 (0.4)	0.45 (0.2)	0.43 (0.3)	0.253	0.063
Max flexion	−0.60 (0.3)	−0.43 (0.3) ^#^	−0.45 (0.3)	−0.44 (0.3) ^#^	−0.45 (0.3) ^#^	0.006	0.187
Hip power (W/kg)							
Max generation during preswing	1.00 (0.5)	0.92 (0.5)	1.01 (0.8)	0.86 (0.4)	0.95 (0.5)	0.569	0.030
Max absorption during stance	−0.63 (0.4)	−0.66 (0.5)	−0.71 (0.8)	−0.61 (0.5)	−0.73 (0.5)	0.751	0.022

Notes: **^#^** indicates *p* < 0.05, significant difference from barefoot.

**Table 4:** Statistical results for PPP with and without insoles (mean (SD); Unit: kPa).

Plantar Region	Gait Phase	Barefoot	PORON^®^ Medical 4708	Pe-Lite	Nora Lunalight A Fresh	Nora Lunalastik EVA	*p* Value	$\eta_{p}^{2}$
Toes	Stance	243.87 (107.3)	227.90 (61.7)	265.37 (48.6)	243.23 (54.7)	312.69 (81.7) ^#^	0.002	0.248
	Swing	56.10 (65.8)	38.22 (14.5)	40.90 (25.5)	50.60 (55.1)	41.55 (18.9)	0.491	0.036
Forefoot	Stance	350.50 (82.03)	180.04 (40.9) **	229.39 (67.3) **	249.79 (85.7) **	199.03 (46.7) **	<0.001	0.705
	Swing	41.86 (12.1)	33.91 (12.8)	31.66 (10.9)	46.30 (38.0)	36.31 (19.9)	0.172	0.084
Midfoot	Stance	142.34 (58.6)	103.30 (27.5)	131.72 (37.9)	128.05 (33.6)	106.10 (30.6)	0.009	0.191
	Swing	26.79 (10.3)	29.13 (9.0)	29.05 (5.6)	34.33 (17.4)	31.43 (12.5)	0.253	0.066
Rearfoot	Stance	303.48 (68.7)	161.48 (19.6) **	201.71 (27.6) **	220.44 (32.0) **	176.11 (16.4) **	<0.001	0.738
	Swing	144.29 (50.5)	61.19 (21.0) **	67.50 (21.5) **	81.91 (40.2) ^#^	68.69 (25.6) **	<0.001	0.559

Notes: **^#^** indicates *p* < 0.05, ** *p* < 0.001, significant difference from barefoot.

## Data Availability

Data are available upon reasonable request.
